# Enhancing HMM-based protein profile-profile alignment with structural features and evolutionary coupling information

**DOI:** 10.1186/1471-2105-15-252

**Published:** 2014-07-25

**Authors:** Xin Deng, Jianlin Cheng

**Affiliations:** LexisNexis | Risk Solutions | Healthcare, Orlando, FL 32811 USA; Computer Science Department, Informatics Institute, C. Bond Life Science Center, University of Missouri-Columbia, Columbia, MO 65211 USA

## Abstract

**Background:**

Protein sequence profile-profile alignment is an important approach to recognizing remote homologs and generating accurate pairwise alignments. It plays an important role in protein sequence database search, protein structure prediction, protein function prediction, and phylogenetic analysis.

**Results:**

In this work, we integrate predicted solvent accessibility, torsion angles and evolutionary residue coupling information with the pairwise Hidden Markov Model (HMM) based profile alignment method to improve profile-profile alignments. The evaluation results demonstrate that adding predicted relative solvent accessibility and torsion angle information improves the accuracy of profile-profile alignments. The evolutionary residue coupling information is helpful in some cases, but its contribution to the improvement is not consistent.

**Conclusion:**

Incorporating the new structural information such as predicted solvent accessibility and torsion angles into the profile-profile alignment is a useful way to improve pairwise profile-profile alignment methods.

## Background

Pairwise protein sequence alignment methods have been essential tools for many important bioinformatics tasks, such as sequence database search, homology recognition, protein structure prediction and protein function prediction [1–5]. Following the development of global and local alignment methods of aligning two single sequences [6–8], profile-sequence alignment or profile-profile alignment methods such as PSI-BLAST, SAM [9], HMMer [10], HHsearch, HHsuite [4–6], which enrich two single sequences with their homologous sequences, has substantially improved both the sensitivity of recognizing remote homologs and the accuracy of aligning two protein sequences.

Due to their relatively high sensitivity in recognizing remote protein homologs, profile-profile alignment methods have become the default structural template identification method for many template-based protein structure modeling methods and servers [11–14]. For instance, HHsearch, one of top profile-profile alignment tools based on comparing the profile hidden Markov models (HMM) of two proteins, was used by almost all the template-based protein structure prediction methods tested during the last two Critical Assessment of Techniques for Protein Structure Prediction (CASP) [15, 16]. The open source package HHsuite contains both the latest implementation of HHSearch that supports a full HMM-HMM alignment-based search on a HMM profile database and a very fast search tool HHblits [5] that reduces the number of unnecessary full HMM pairwise alignment in order to drastically improve its search speed. Moreover, the maximum accuracy (MAC) alignment algorithm is applied in HHsuite, but not in HHsearch. In this work, we aim to introduce new sources of information to improve profile-profile alignments with respect to both the original HHsearch package and the open source HHsuite package,

In order to more accurately align the structurally equivalent residues in a target protein and a template protein together, secondary structure information was incorporated into profile-profile sequence alignment methods, yielding the better sensitivity and accuracy [4, 17]. Aiming to find the new source of information to further improve the sensitivity and accuracy of pairwise profile-profile alignment, we examine the effectiveness of incorporating into profile-profile alignment methods some new features that have not been used in profile-profile alignments before, including protein solvent accessibility, torsion angles, and the evolutionary residue coupling information [18, 19].

Specifically, we add the additional scoring terms for solvent accessibility, torsion angles, and evolutionary residue coupling information into the scoring function of HHsuite [5] in order to enhance the alignment process. According to our evaluation, adding solvent accessibility and torsion angles can improve the alignment accuracy, but incorporating the evolutionary residue coupling information is only useful in some cases.

## Methods

**Figure 1:**
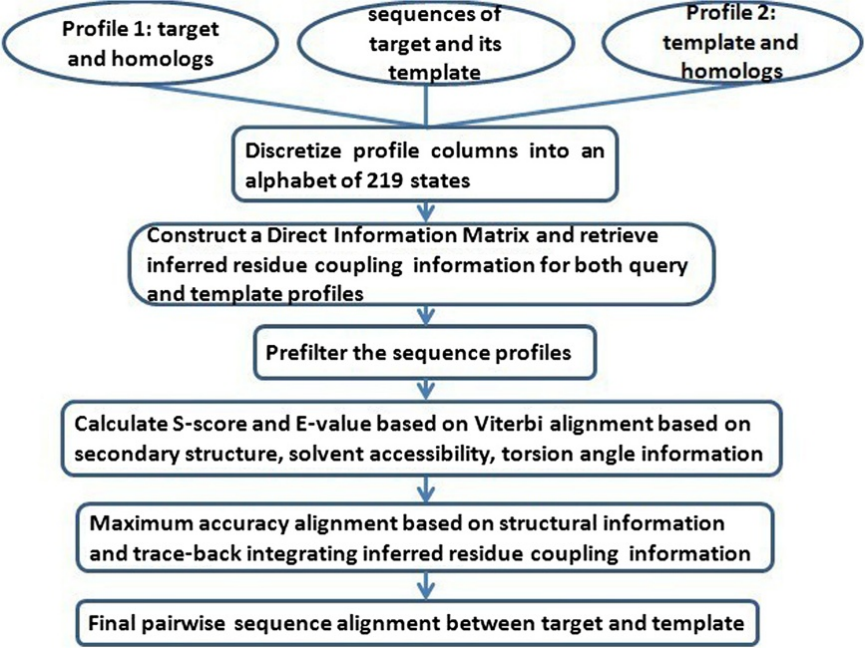
**The workflow of the HMM-based profile-profile pairwise alignment.**

We extended an existing profile-profile alignment method within the standard five-step alignment framework of HHsuite [5] shown in Figure 1, including discretization of profile columns, removal of very short or very dissimilar sequences, execution of Viterbi alignment and calculation of E-value and probability, realignment based on the maximum accuracy (MAC) algorithm, and retrieval of alignments by tracing-back. Different from HHsuite, our method applies solvent accessibility and torsion angle information to both the Viterbi alignment and the maximum accuracy alignment, and traces back with the aid of the evolutionary residue coupling information. In the following sections, we focus on describing how to incorporate the new features into the profile-profile method (i.e., HHsuite), while briefly introducing the necessary technical background.

### Adding solvent accessibilities and torsion angles into the viterbi alignment

The score of aligning two columns in two protein profiles (namely a query profile *q* and a template profile *t*) in HHsuite was calculated according to Equation (1).
1

in which *q*_*i*_(*a*) and *t*_*j*_(*a*) denote the probability of amino acid at position *i* in the query profile and at position *j* the template profile, respectively, and *f*(*a*) is the background frequency of residue *a* (*a* ∈ {1, 2,...., 20}, representing 20 types of amino acids). The best alignment between two profile HMMs was obtained by maximizing the log-sum-odds score *S*_*LSO*_ according to Equation (2).
2

where *k* denotes the index of columns that query HMM *q* aligned to template HMM *t*, *i*(*k*) and *j*(*k*) are the respective columns in *q* and *t*, *P*_*tr*_ is the product of all transition probabilities for the path through *q* and *t*. The latest version of HHsuite has included the secondary structure information into the calculation of the score. In this work, we further augment the calculation of the score by adding the terms to account for the solvent accessibility, and torsion angles.

The Viterbi dynamic program algorithm used five matrices *S*_*AB*_ (i.e., *AB* ∈ {*MM*, *MI*, *IM*, *DG*, *GD*}) representing matching different states (M: match, I: insertion, D: deletion; G: Gap [4]) in two HMMs to maximize the augmented log-sum-of-odds score *S*_*LSO*_. They are recursively calculated as:
345

*S*_*IM*_(*i*, *j*) and *S*_*GD*_(*i*, *j*) are calculated similarly as *S*_*MI*_(*i*, *j*) and *S*_*DG*_(*i*, *j*).

The difference between Equation (3) above and the default one in HHsuite is that two new terms (*S*_sa_, *S*_tors_) were added to utilize the solvent accessibility and torsion angle information. In Equation (3), *S*_*ss*_(*q*_*i*_, *t*_*j*_) is the secondary structure score between column *i* in query HMM (*q*_*i*_) and column *j* in template HMM (*t*_*j*_), which was the same as the one originally used in HHsuite. *S*_*sa*_(*q*_*i*_, *t*_*j*_) is the solvent accessibility score between *q*_*i*_ and *t*_*j*_, and *S*_*tors*_(*q*_*i*_, *t*_*j*_) is the torsion angle score between *q*_*i*_ and *t*_*j*_, which are the new terms introduced in this work. *w*_*ss*_, *w*_*sa*_, and *w*_*tors*_ are weights for the secondary structure score, solvent accessibility score and torsion angle score respectively. *S*_*shift*_ is the score offset for match-match states. Three weights *w*_*ss*_, *w*_*sa*_, *w*_*tors*_ and shift score *S*_*shift*_ are set to 0.11, 0.72, 0.4 and −0.03 by default, and can be adjusted by users as well. *q*_*i* − 1_(*M*, *M*) is the transition probability from state M at column *i-1* to next state M of in the query HMM, and *t*_*j* − 1_(*M*, *M*) is the transition probability from state M at column *j-1* to next state M in the template HMM.

Here we denote this extension of the HHsuite method as HMMsato. HMMsato allows for scoring predicted (or known) solvent accessibilities of one protein against predicted (or known) ones of another protein. DSSP [20] is used to parse the true solvent accessibility of a protein if its tertiary structure is known. PSpro 2.0 [21] is used to predict the solvent accessibility of a protein. The solvent accessibility information can be automatically parsed or predicted in HMMsato, or alternatively provided by a user. The two types of solvent accessibilities (e: exposed, > = 25% of the maximum area of a residue is exposed; b: buried, < 25% of the maximum area of a residue is exposed) are employed. Assuming the predicted or true solvent accessibility states of the i^th^ residue (*x*_*i*_) of the query protein and the j^th^ residue (*y*_*j*_) of the template protein are *sa*(*x*_*i*_) and *sa*(*y*_*j*_), the solvent accessibility score between the two residues *S*_*sa*_(*q*_*i*_, *t*_*j*_) is defined as:
6

The score is calculated by the kronecker-delta function *δ*(*a*, *b*), which equals to 1 if *a* = *b*, 0 otherwise.

Similarly as the solvent accessibility, the torsion angles including both phi angle (*φ*) and psi angle (*ψ*) can be automatically predicted by SPINE-X [22, 23] or provided by a user. The range of both *φ* and *ψ* is (−180,180). Given the query sequence X and template sequence Y, the predicted phi angle and psi angle of the i-th residue *x*_*i*_ in the query are denoted as *φ*(*x*_*i*_) and *ψ*(*x*_*i*_), and those of the *j*-th residue *y*_*j*_ in the template as *φ*(*y*_*j*_) and *ψ*(*y*_*j*_). The torsion angle score *S*_*tors*_(*q*_*i*_, *t*_*j*_) between the two residues is calculated as:
7

### Realign the profiles by maximum accuracy alignment combining solvent accessibility and torsion angles

It has been shown that maximum accuracy (MAC) algorithm can generally create a more accurate alignment than the Viterbi algorithm, while the latter can generate better alignment scores, e-values and probabilities [5, 24]. Consequently, the Viterbi algorithm is applied to compute e-values and scores, and the MAC algorithm is chosen to generate the final HMM-HMM pairwise alignment in HHsato by default.

The maximum accuracy algorithm [5, 24] creates the local alignment that maximizes the sum of probabilities for each residue pair to be aligned minus a penalty (*mact*) (i.e., *argmax*() ), where  represents the posterior probability of the match state *i* in HMM *q* aligned to the match state *j* in HMM *t*. With the parameter *mact*, users can control the alignment greediness, from nearly global, long alignment (*mact* = 0) to very precise, short local alignments (*mact* ≈ 1). The default value of *mact* is set to 0.3501 in HMMsato as in HHsuite. To find the best MAC alignment path, an optimal sub-alignment score matrix *AS* is calculated recursively using the posterior probability  as substitution scores:
8

Here, the Forward-Backward algorithm in local or global mode is applied to calculate the posterior probabilities . The Forward partition function *F*_*MM*_(*i*, *j*) and Backward partition function *B*_*MM*_(*i*, *j*) are introduced to calculate the posterior probability for pair state () according to Equation (9):
9

Five dynamic programming matrices *F*_*AB*_ are used to compute the Forward partition function *F*_*MM*_, and *AB* ∈ {*MM*, *MI*, *IM*, *DG*, *GD*}. The top row and left column of the *F*_*MM*_ matrix were initialized to 0, and all the matrices were filled recursively:
10

where *p* min controls the alignment model (0: global alignment mode, 1: local alignment mode). *F*_*IM*_(*i*, *j*) and *F*_*GD*_(*i*, *j*) are calculated similarly as *F*_*MI*_(*i*, *j*) and *F*_*DG*_(*i*, *j*). Solvent accessibility score *S*_*sa*_(*q*_*i*_, *t*_*j*_) and torsion angle score *S*_*tors*_(*q*_*i*_, *t*_*j*_) are calculated as in the Viterbi alignment.

In analogy to the Forward partition function, the Backward partition function matrix *B*_*MM*_ are calculated recursively as follows:
11

*B*_*IM*_(*i*, *j*) and *B*_*GD*_(*i*, *j*) are calculated similarly as *B*_*MI*_(*i*, *j*) and *B*_*DG*_(*i*, *j*).

### Trace back maximum accuracy alignments with the evolutionary residue coupling information

The Evolutionary Coupling (EC) stands for the correlation between two positions or columns in a multiple protein sequence alignment or a protein profile [19, 20]. It has recently been employed to predict residue-residue contacts [18, 19]. In order to improve profile-profile alignment with the evolutionary coupling information, we calculate the mutual information (MI) (one way of calculating EC value) for any two columns (*i*, *j*) of each profile according to Equation (12).
12

*N* is 21, standing for 20 amino acids plus gap. The joint probability of two residues *X*_*i*_ and *X*_*j*_ (*F*_*ij*_(*X*_*i*_, *X*_*j*_)) and the probability of residue *X*_*i*_ (*F*_*i*_(*X*_*i*_)) are calculated in the same way as in [10]. However, *EC*_*ij*_ is calculated as the mutual information (MI) instead of the direct information (DI) based on the global probability model [19] in order to achieve the higher time efficiency. A higher EC value corresponds to a stronger correlation between two columns in the given profile.

Based on the calculated EC value matrices for both the query and template profiles, top highly correlated position pairs with higher EC values for each profile are selected. The evolutionary residue coupling information is then applied to check the counterpart pairs during the process of tracing back through the sub-alignment score matrix *AS* (see Equation (8)) of the MAC alignment. Specifically, we denote the evolutionary coupled position for position *i* in query *q* as *k*_*q*_(*i*), and the coupled position of position *j* in template *t* as *k*_*t*_(*j*). Moreover, *M*_*q*_(*i*) denotes the position in template *t* matched with position *i* in query *q* when tracing back the original *AS* matrix, *M*_*t*_(*j*) denotes the position in query *q* matched with position *j* in template *t* when tracing back the original *AS* matrix, and *w*_*ec*_ is the weight for the evolutionary coupling information. The new *AS*^'^ matrix integrating the evolutionary coupling information is recalculated as follows during the track back process.
13

**Figure 2:**
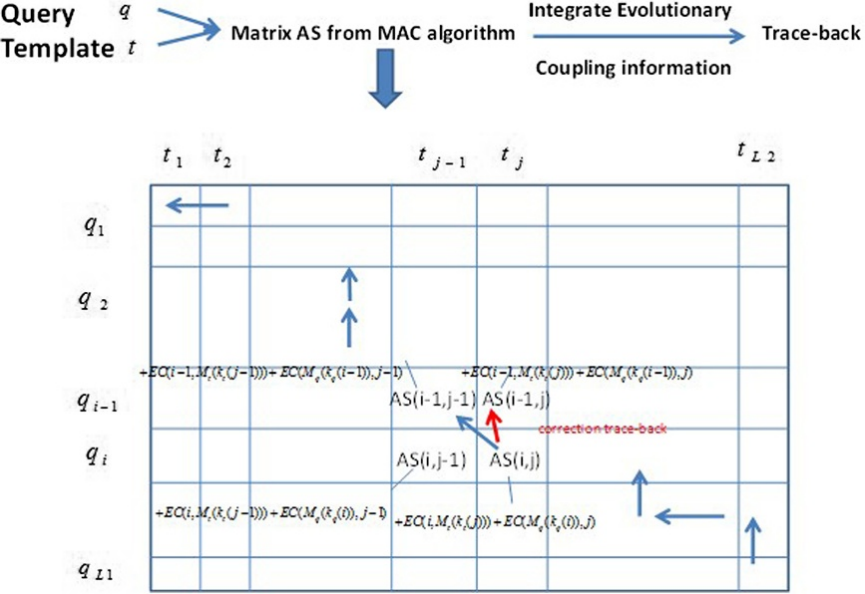
**Tracing back from the** ***AS*** **matrix by integrating the evolutionary coupling information.** In query q, the coupled position of *i* is *k*_*q*_(*i*)_,_ and that of *i-1* is *k*_*q*_(*i* − 1). In template t, the coupled position of *j* is *k*_*t*_(*j*)_,_ and that of *j-1* is *k*_*t*_(*j* − 1). *M*_*q*_(*i*) is the corresponding position in template t matched to position *i* in q during the original tracing-back. *M*_*t*_(*j*) is the corresponding position in query q matched to position *j* in t during the original tracing-back. Additional EC scores are added into the corresponding elements in the *AS* matrix as shown in the figure so that the correct tracing back is performed.

Figure 2 illustrates an exampling of taking into account the evolutionary coupling information during the tracing back process to generate the final alignment.

## Results and discussion

### Evaluation data set and metric

We evaluated HMMsato along with HHSearch [4] and HHsuite on the alignments between 106 targets (queries) of the 9^th^ Critical Assessment of Techniques for Protein Structure Prediction (CASP9) [15, 16] and their homologous template proteins (templates) released at the CASP9’s web site. The alignment data set has 2,621 pairs of query and template proteins. 1,483 pairs associated with 60 CASP9 targets were used as optimization data set to optimize the parameters of HMMsato, and 1,138 pairs associated with the remaining 46 CASP9 targets were used to test the methods. The reference (presumably true) pairwise alignments of a query-template protein pair was generated by using TMalign [25] to align the tertiary (3D) structures of the two proteins together. The alignments generated by HMMsato and other tools were evaluated by three metrics, including sum-of-pairs (SP) score, true column (TC) score, and the quality of the tertiary structural models of the query proteins built from the alignments. The SP and TC scores are the two standard metrics for evaluating sequence alignment quality [26]. The quality of tertiary structural models indirectly assesses the quality of sequence alignments according to their effectiveness in guiding the construction of protein structural models.

The SP score is the number of correctly aligned pairs of residue in the predicted alignment divided by the total number of aligned pairs of residues in the core blocks (i.e., sequence alignment regions precisely determined by structural alignment of structurally equivalent residues in the structures of two proteins) of the true alignment [23]. The TC score is the number of correctly aligned columns in the core blocks of the true alignment [27]. The 3D model of a query protein was produced by MODELLER [28] based on both the pairwise alignment generated by an alignment method and the known structure of the template protein in the alignment. We used TM-Score [29] to align a 3D model of a query protein against its true structure to generate TM-scores and GDT-TS scores [30] for the model in order to measure the quality of the alignment used to generate the model, assuming better alignments lead to better 3D models with higher TM-scores and GDT-TS scores. Both TM-score and GDT-TS score are in the range [0, 1] [31].

### Optimization of weights for the solvent accessibility, torsion angles and evolutionary coupling information

We estimated the weights of the solvent accessibility, torsion angles and evolutionary residue coupling information on the training alignments step by step. Firstly, we found the best weight value (*w*_*sa*_ = 0.72) for solvent accessibility. Then, we identified the best weight value (*w*_*tors*_ = 0.4) for torsion angles while keeping the weight for solvent accessibility fixed. Finally, we found the best parameter value (*w*_*ec*_ = 0.1) for the evolutionary residue coupling information by keeping *w*_*sa*_ and *w*_*tors*_ at their optimum values. HHsearch and HHsuite were both evaluated with and without secondary structure information. The default parameter values were used with HHsearch and HHsuite.

### Comparison of HMMsato, HHSearch, and HHsuite on the test data set

The mean SP and TC scores for the pairwise alignment results generated by HMMsato, HHSearch and HHsuite for 1,138 protein pairs are reported in Table 1. The mean SP score and the mean TC score of HMMsato are 50.39 and 50.02 respectively, higher than HHsearch and HHsuite with or without secondary structure information. The average TM-scores and GDT-TS scores of the 3D models successfully generated from 1,127 out of 1,138 alignments by MODELLER were listed in Table 2. The average TM-score and GDT-TS score of the models generated from the HMMsato alignments are 0.555 and 0.483, respectively, better than those of HHSearch and HHsuite. Furthermore, we carried out the Wilcoxon matched-pair signed-rank test on both SP and TC scores of the three methods on the test data set. The p-values of alignment score differences between HMMsato and the other methods calculated by the Wilcoxon matched-pair signed-rank test are reported in Table 3.

**Table 1:** **The mean SP and TC scores of the pairwise alignments generated by HHsearch1.2, HHsuite and HMMsato on the CASP9 test data set consisting of 1,138 pairs of proteins**

Method	Mean SP score	Mean TC score
HHsearch (without secondary structure information)	48.69	48.34
HHsearch (with secondary structure information)	50.00	49.65
HHsuite (without secondary structure information)	48.47	48.12
HHsuite (with secondary structure information)	49.76	49.41
HMMsato	**50.39**	**50.02**

Bold numbers are the highest scores.

**Table 2:** **The average TM-scores and GDT-TS scores of the 3D models generated from the 1,127 pairwise test alignments produced by HHsearch1.2, HHsuite and HMMsato**

Method	Average TM-score	Average GDT- TS score
HHsearch (without secondary structure information)	0.527	0.459
HHsearch (with secondary structure information)	0.548	0.479
HHsuite (without secondary structure information)	0.525	0.459
HHsuite (with secondary structure information)	0.543	0.476
HMMsato	**0.555**	**0.483**

Bold numbers are the highest scores.

**Table 3:** **The statistical significance (p-values) of SP and TC score differences between HMMsato and the other two tools on the test data set**

Tools	p-value of SP scores	p-value of TC scores
HMMsato -- HHsearch (without secondary structure information)	1.078 X 10^−6^	3.414 X 10^−7^
HMMsato -- HHsearch (with secondary structure information)	0.7538	0.8082
HMMsato -- HHsuite (without secondary structure information)	1.724 X 10^−8^	1.515 X 10^−9^
HMMsato -- HHsuite (with secondary structure information)	0.1535	0.1087

### Impact of solvent accessibility, torsion angles and evolutionary coupling information on the alignment accuracy

**Figure 3:**
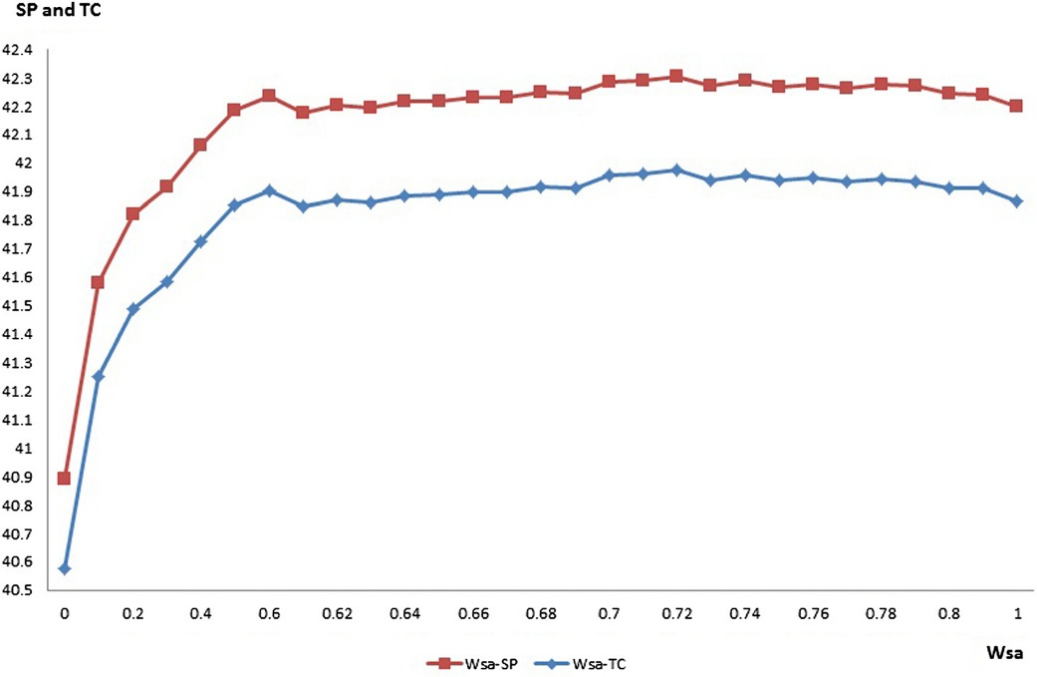
**The plot of the SP and TC scores against different values of the weight of solvent accessibility (w**_**sa**_ **).**

We studied the effect of the solvent accessibility information by solely adjusting the value of its weight w_sa_. The SP scores and TC scores of the alignments generated by HMMsato with different w_sa_ values on the training data set are shown in Table 4. The results show that incorporating the solvent accessibility information always improves alignment accuracy in comparison with the baseline not using solvent accessibility information (w_sa_ = 0). The highest accuracy is achieved when w_sa_ is set to 0.72. Figure 3 shows the plot of SP scores/TC scores against the different values of w_sa._ Red curve represents the SP scores and blue represents the TC scores.

**Table 4:** **The SP scores and TC scores with different values of w**_**sa**_ **using HMMsato on the training data**

***w***_***sa***_	***0***	***0.1***	***0.2***	***0.3***	***0.4***	***0.5***	***0.6***	***0.61***	***0.62***
SP score	40.89	41.58	41.82	41.92	42.06	42.18	42.23	42.18	42.20
TC score	40.58	41.25	41.49	41.58	41.73	41.85	41.90	41.85	41.87
*0.63*	*0.64*	*0.65*	*0.66*	*0.67*	*0.68*	*0.69*	*0.7*	*0.71*	*0.72*
42.19	42.22	42.22	42.23	42.23	42.25	42.24	42.29	**42.29**	**42.31** ^*^
41.86	41.89	41.89	41.90	41.90	41.92	41.91	41.96	**41.96**	41.98*
*0.73*	*0.74*	*0.75*	*0.76*	*0.77*	*0.78*	*0.79*	*0.8*	*0.9*	*1*
42.27	42.29	42.27	42.28	42.27	42.28	42.27	42.25	42.24	42.20
41.94	41.96	41.94	41.95	41.94	41.94	41.94	41.91	41.91	41.87

Bold denotes the two best scores, and an extra superscript of star denotes the highest score.

**Figure 4:**
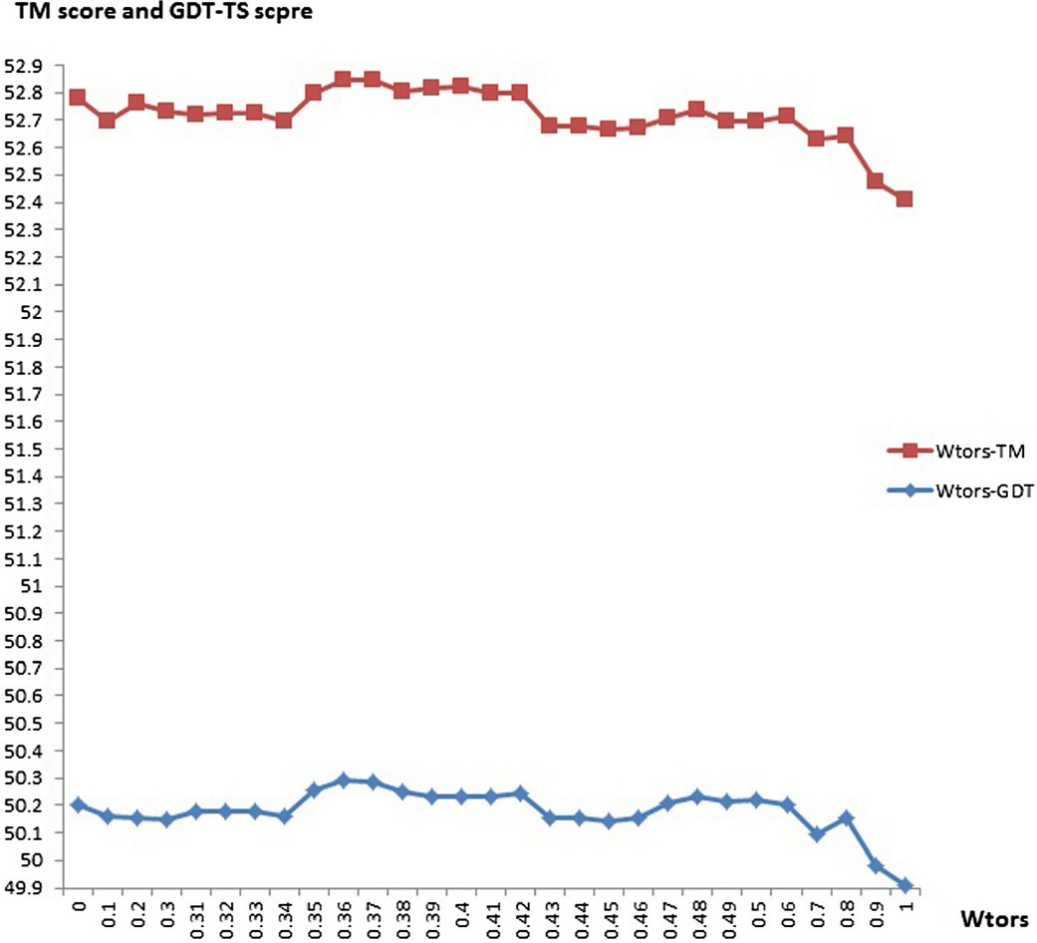
**The plot of the TM-scores and GDT-TS-scores against different values of the weight of torsion angles (w**_**tors**_ **).**

We studied the effect of torsion angles on alignments by solely adjusting the value of w_tors_ (weight for torsion angle information) while keeping w_sa_ as 0.72. The SP scores and TC scores of the alignments generated by HMMsato with different w_tors_ values on the training data set are shown in Table 5. The results show that incorporating the torsion angle information also helps improve alignment accuracy. The highest accuracy is achieved when w_tors_ is set to 0.4. Figures 4 shows the TM-scores and GDT-TS scores of the 3D models constructed from the alignments generated by HMMsato with both torsion angles and solvent accessibility with respect to different w_tors_ values.

**Table 5:** **The SP scores and TC scores with different values of w**_**tors**_ **using HMMsato**

***W***_***tors***_	***0***	***0.1***	***0.2***	***0.3***	***0.31***	***0.32***	***0.33***	***0.34***	***0.35***
SP score	42.31	42.32	42.35	42.45	42.47	42.47	42.47	42.49	42.50
TC score	41.98	41.99	42.02	42.12	42.14	42.14	42.14	42.16	42.16
*0.36*	*0.37*	*0.38*	*0.39*	*0.4*	*0.41*	*0.42*	*0.43*	*0.44*	*0.45*
42.50	42.51	42.50	42.51	**42.53** ^*^	**42.52**	42.49	42.50	42.50	42.51
42.17	42.17	42.17	42.18	**42.19** ^*^	**42.19**	42.15	42.16	42.17	42.17
*0.46*	*0.47*	*0.48*	*0.49*	*0.5*	*0.6*	*0.7*	*0.8*	*0.9*	*1*
42.51	42.50	42.50	42.50	42.50	42.46	42.45	42.40	42.46	42.40
42.17	42.16	42.17	42.17	42.17	42.13	42.12	42.07	42.13	42.07

Bold denotes the two best scores, and an extra superscript of star denotes the highest score.

### The effect of evolutionary residue coupling information on alignment accuracy

We studied the effect of the evolutionary residue coupling information on alignment accuracy in a similar way. HMMsato worked the best when *w*_*ec*_ was 0.1. However, the evolutionary coupling information did not improve the overall alignment accuracy on the training data set, probably due to lack of a large number of diverse sequences in many cases required by the evolutionary coupling calculation to obtain the sufficient discriminative power. Specifically speaking, the alignment quality increased in 57 alignments, stayed the same in 1363 alignments, but decreased in 61 alignments. Similarly, on the test data set, the alignment quality increased in 59 alignments, stayed the same in 1024 alignments, but decreased in 55 alignments. Generally speaking, the evolutionary coupling information contributed to the improvement of alignment accuracy in some cases, but its effect was rather inconsistent.

### Comparison of HMMsato and HHSearch with secondary structure information on the test data set

**Figure 5:**
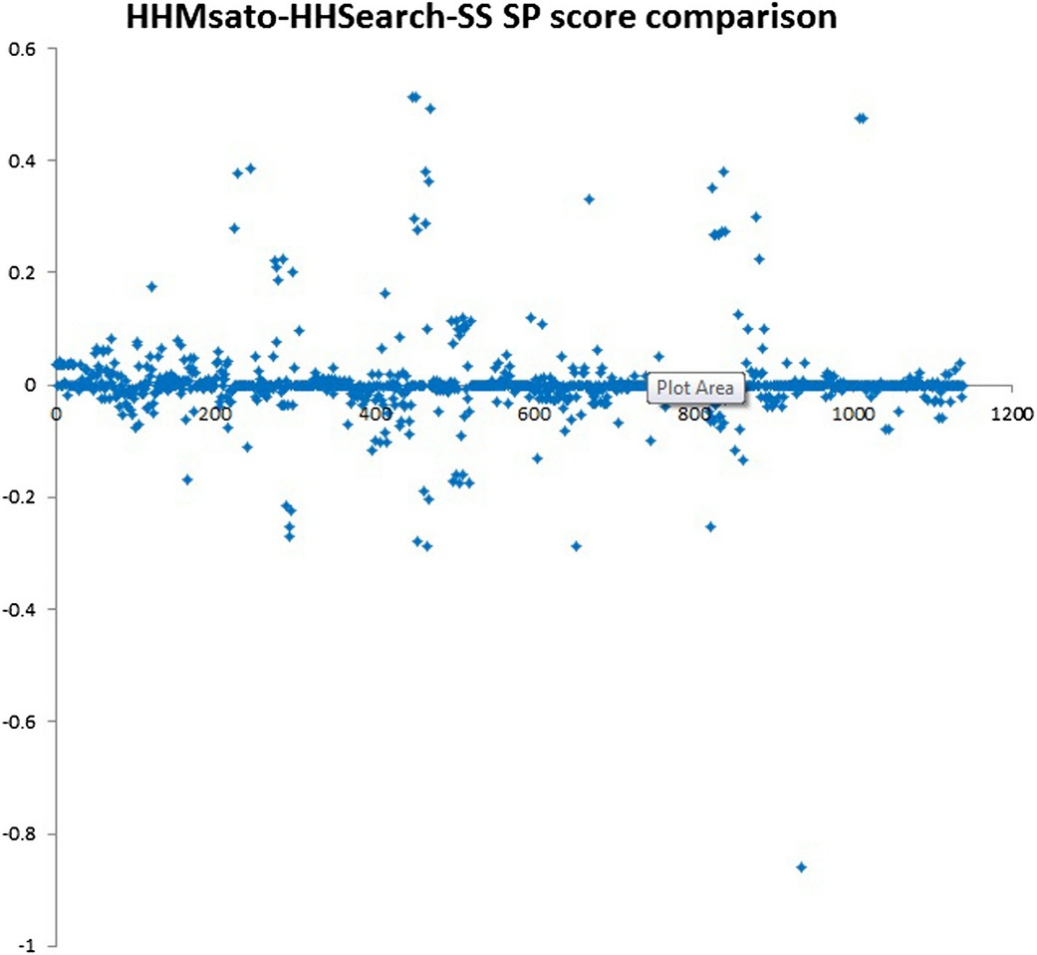
**The plot of the SP score differences between HMMsato and HHsearch with secondary structure (HMMsearch-SS) for all the 1138 testing pairs.** X-axis represents the index of the testing pair (1–1138), and y-ray represents the SP score difference (the SP score of HMMsato – the SP score of HHSearch-SS) for all the testing pairs.

**Figure 6:**
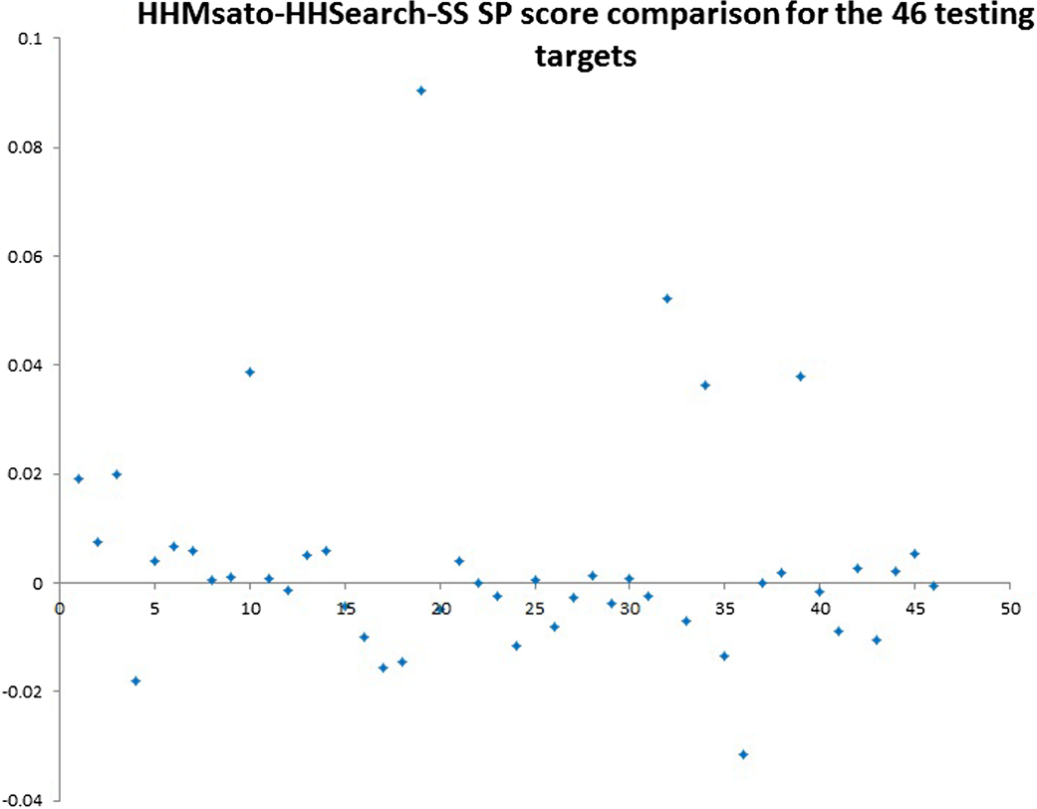
**The plot of the average SP score difference between HMMsato and HHSearch-SS for the 46 testing protein targets.** X-axis represents the index of the testing targets (1–46), and y-axis represents the score difference.

We studied the SP score differences between HMMsato and HHSearch with secondary structure for all the 1138 testing pairs. The plot of the SP score difference (SP score of HMMsato minus SP score of HHSearch) for these pairs is shown in Figure 5. Similarly, the plot of the average SP score difference between HMMsato and HHSearch-SS for the 46 testing protein targets is shown in Figure 6. X-axis represents the index of the testing targets (1–46), and y-axis represents the score difference. Specifically, the alignment quality increased for 24 targets, stayed the same for 2 targets, but decreased for 20 targets. We found that HMMsato often improved the alignment quality for proteins of length ranging from 70 to 450 residues.

## Conclusion

We designed a method to incorporate relative solvent accessibility, torsion angles and evolutionary residue coupling information into HMM-based pairwise profile-profile protein alignments. Our experiments on the large CASP9 alignment data set showed that utilizing solvent accessibility and torsion angles improved the accuracy of HMM-based pairwise profile-profile alignments. However, the effect of the evolutionary residue coupling information on alignments is less consistent according to our current experimental setting, even though it may still be a valuable source of information to explore in the future. Particularly, we will use the latest method (i.e., direct information) of calculating evolutionary coupling information to guide the profile alignment process. Furthermore, we will carry out more extensive search of optimal weights for solvent accessibility, torsion angle, secondary structure, and evolutionary coupling information to improve alignment accuracy.
